# Supplementary material for: Genome-Wide Analysis of the MYB Transcription Factor Superfamily in Physcomitrella patens
Source: Int J Mol Sci. 2020 Feb 1;21(3):975. doi: 10.3390/ijms21030975 (PMC7037163; doi:10.3390/ijms21030975)
Supplement: Supplementary file 1 [file ijms-21-00975-s001.pdf]

Supplementary Figures

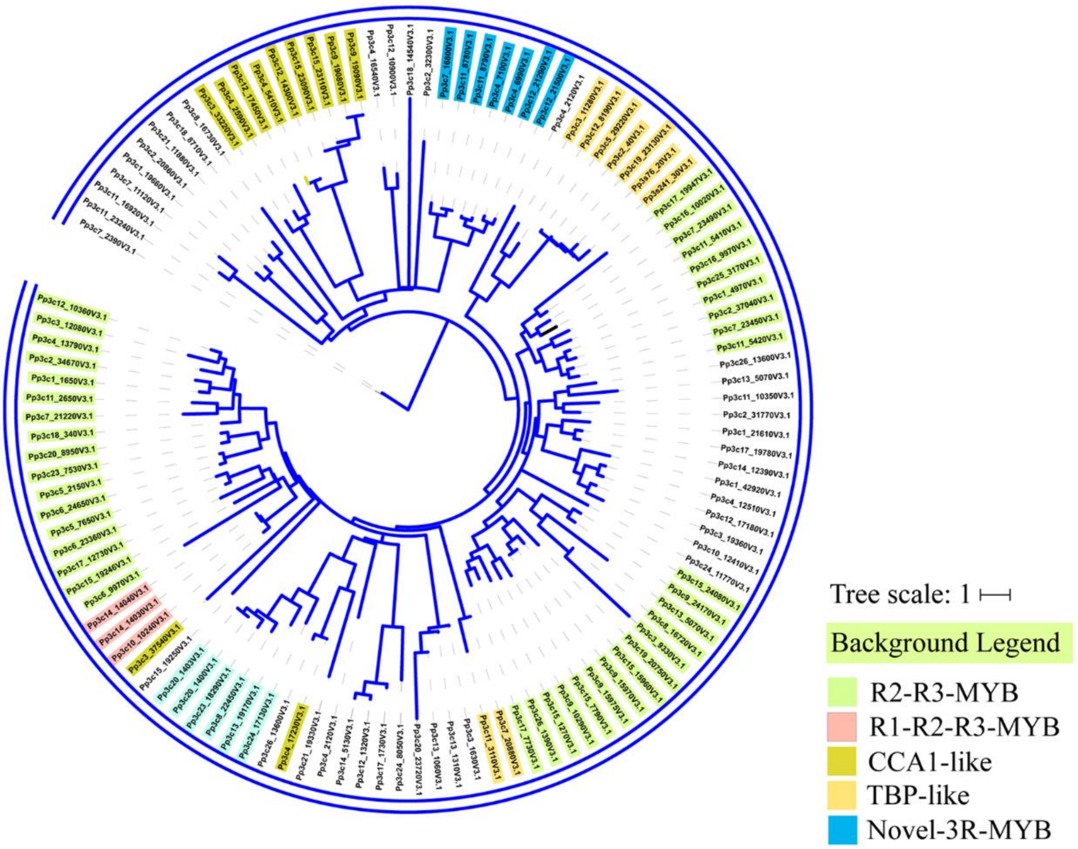

Figure S1. A phylogenetic tree of MYB transcription factors of *Physcomitrella patens*.

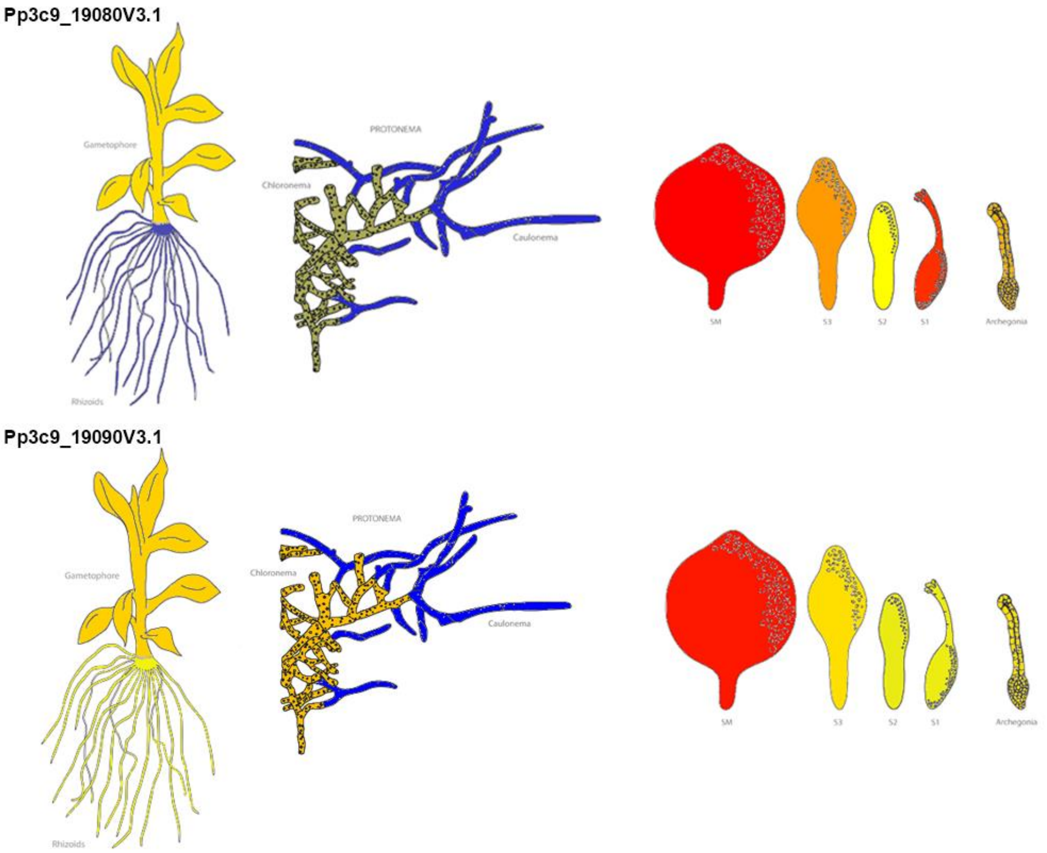

Figure S2. Expression pattern of *Pp3c9_19080* and *Pp3c9_19090*.

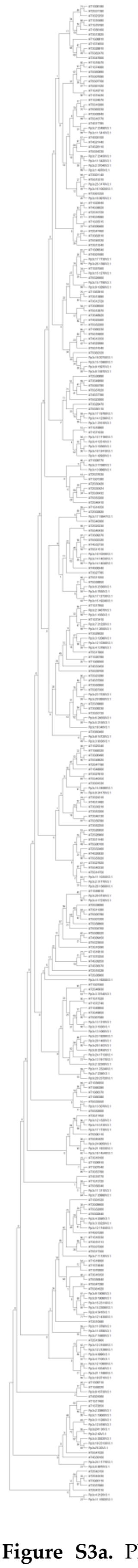

Figure S3a. Phylogenetic analysis of the MYB transcription factors of *Arabidopsis thaliana* and *Physcomitrella patens*.

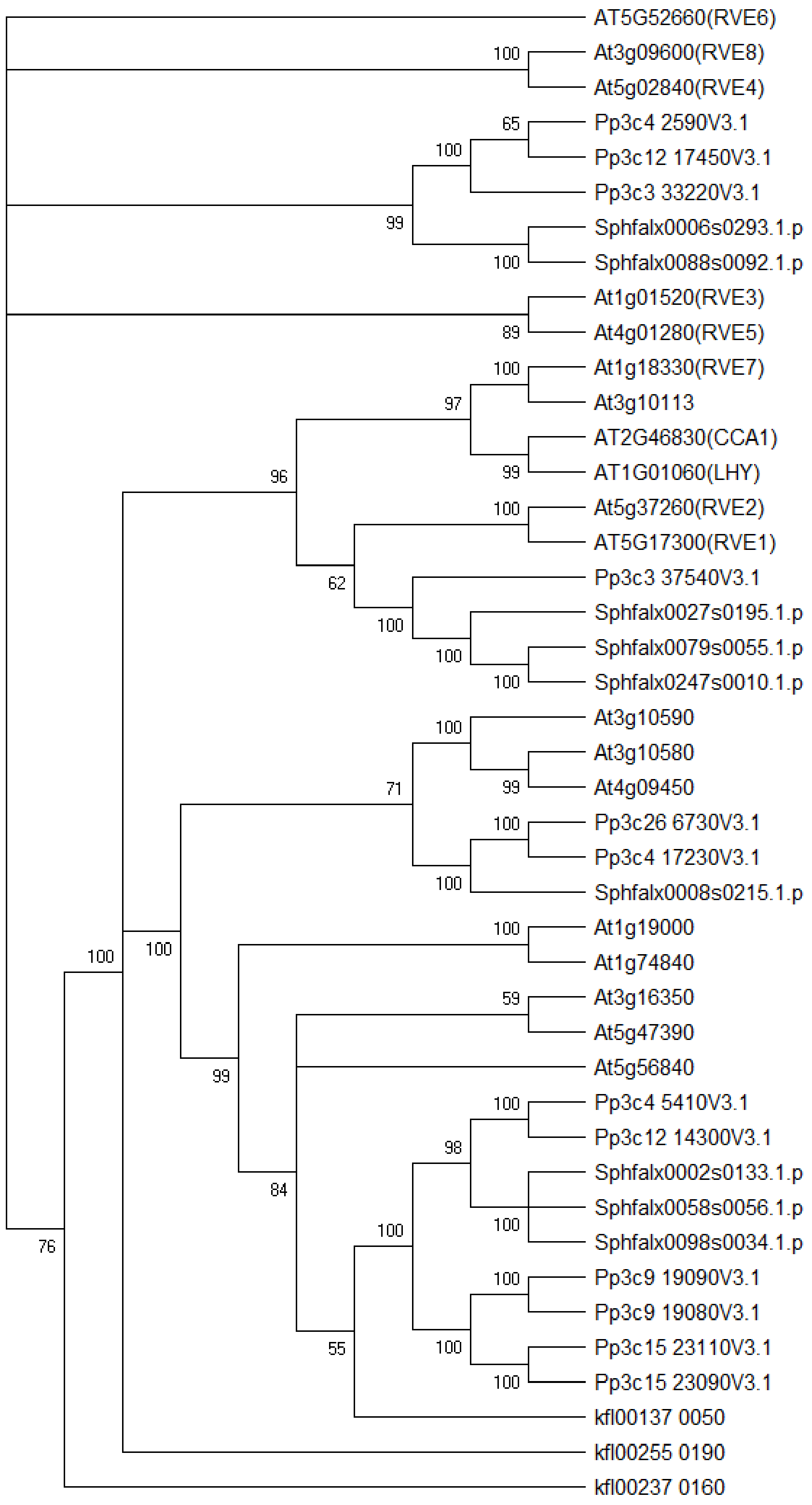

Figure S3b. Phylogenetic analysis of the CCA1-like transcription factors of *Klebsormidium flaccidum*, *Arabidopsis thaliana*, *Sphagnum fallax* and *Physcomitrella patens*.

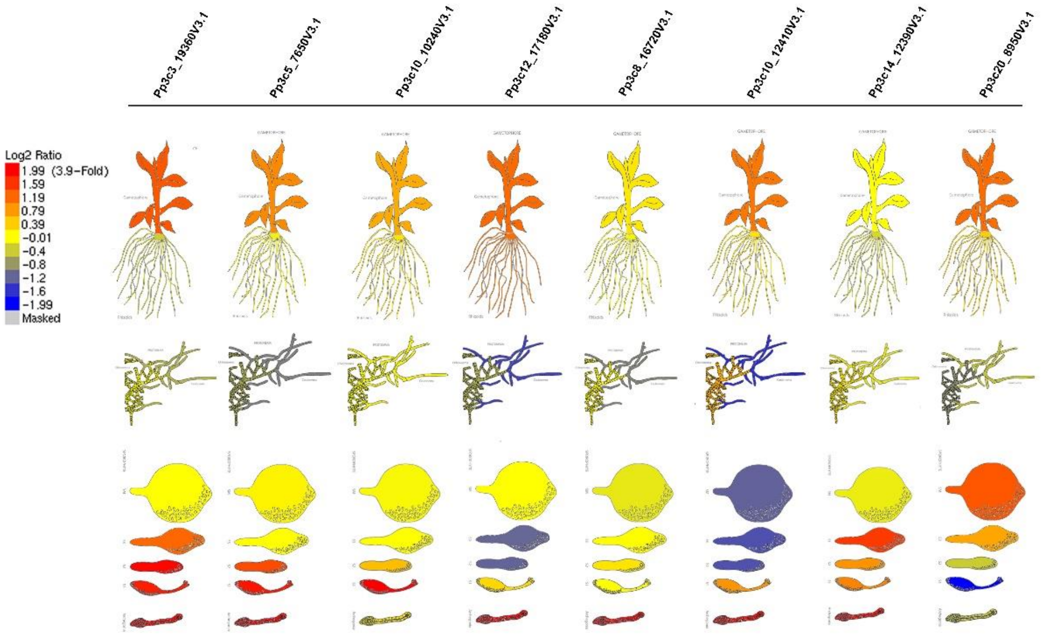

Figure S4a. Expression pattern of R2R3-MYB of *Physcomitrella patens*.

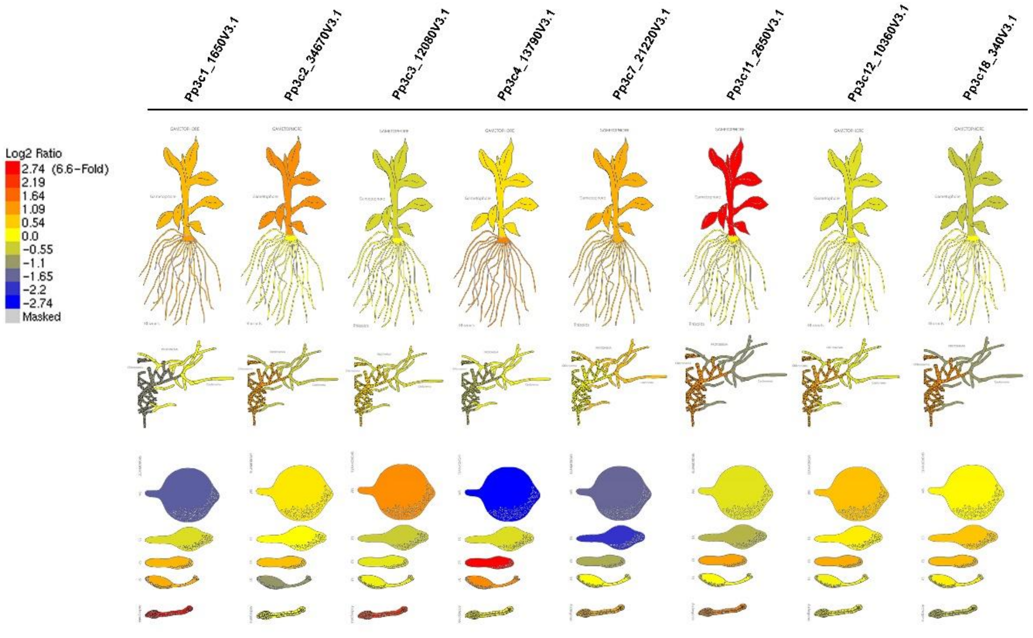

Figure S4b. Expression pattern of R2R3-MYB of *Physcomitrella patens*.

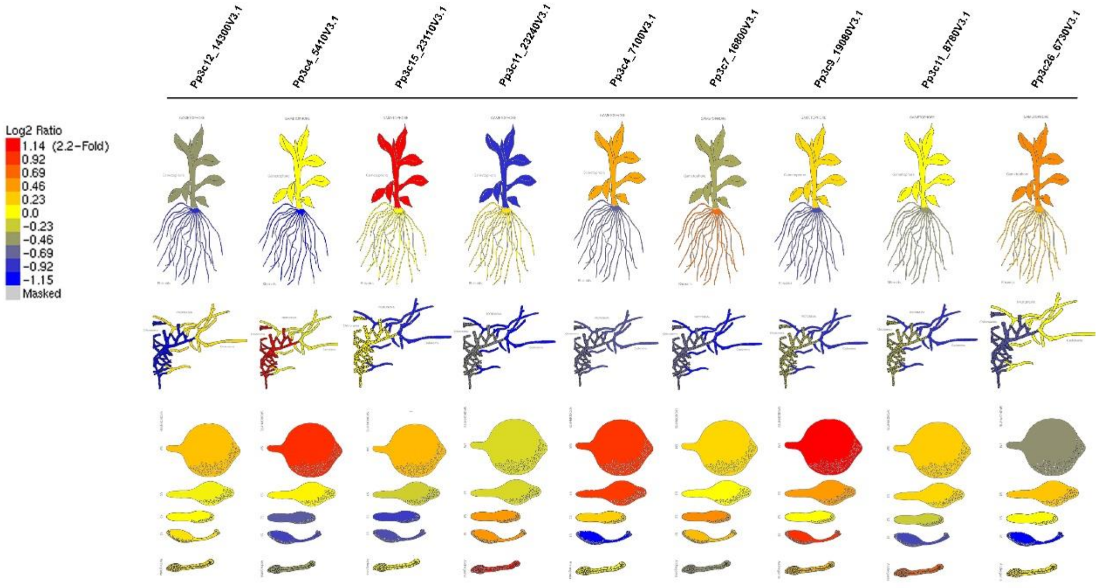

Figure S4c. Expression pattern of CCA1-like of *Physcomitrella patens*.

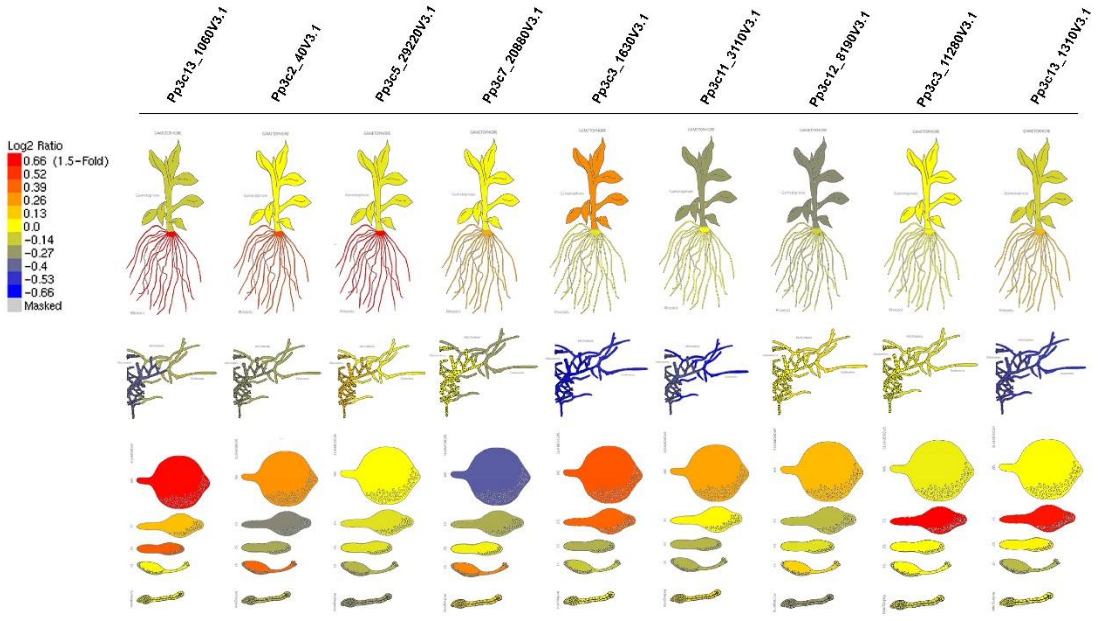

Figure S4d. Expression pattern of TBP-like of *Physcomitrella patens*.

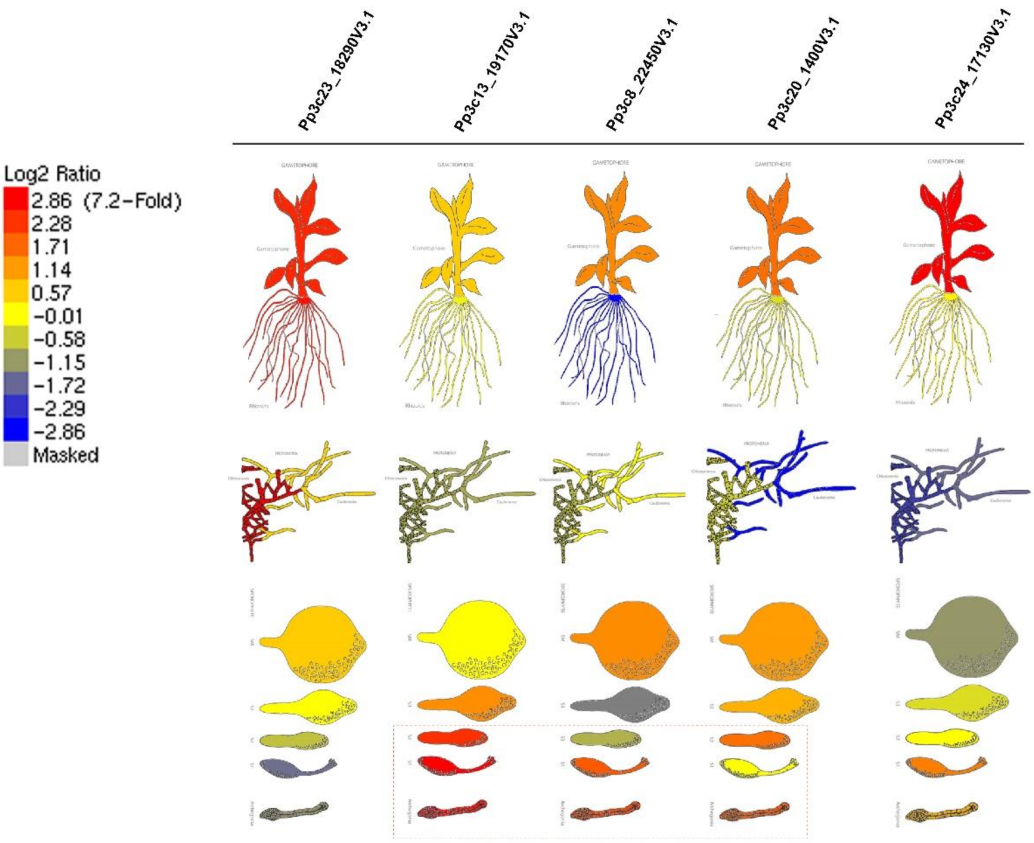

Figure S4e. Expression pattern of bromodomain-containing MYB of *Physcomitrella patens.*

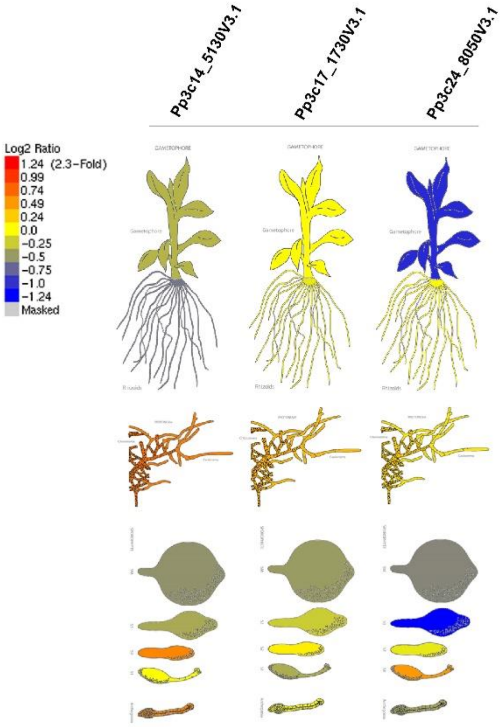

Figure S4f. Expression pattern of DnaJ-domain-containing MYB of *Physcomitrella patens.*

Table S1. Prediction of subcellular localization and analysis of gene duplication of MYB genes.

Gene ID	Cellular localization	Types of gene duplication
Pp3c16_9970V3.1	Nucleus	
Pp3c25_3170V3.1	Nucleus	
Pp3c16_10020V3.1	Nucleus	tandem duplication
Pp3c19_20750V3.1	Nucleus	
Pp3c15_15960V3.1	Nucleus	block duplication
Pp3c9_15975V3.1	Nucleus	tandem duplication
Pp3c9_15970V3.1	Nucleus	tandem duplication
Pp3c7_23490V3.1	Nucleus	tandem duplication and block duplication
Pp3c7_23450V3.1	Nucleus	tandem duplication and block duplication
Pp3c17_19780V3.1	Nucleus	block duplication
Pp3c14_12390V3.1	Nucleus	block duplication
Pp3c11_5420V3.1	Nucleus	tandem duplication and block duplication
Pp3c2_37040V3.1	Nucleus	
Pp3c17_7730V3.1	Nucleus	
Pp3c1_4970V3.1	Nucleus	block duplication
Pp3c11_5410V3.1	Nucleus	tandem duplication and block duplication
Pp3c1_21610V3.1	Nucleus	
Pp3c11_10350V3.1	Nucleus	
Pp3c26_1390V3.1	Nucleus	block duplication
Pp3c2_31770V3.1	Nucleus	
Pp3c15_7790V3.1	Nucleus	block duplication
Pp3c9_10290V3.1	Nucleus	block duplication
Pp3c15_1270V3.1	Nucleus	block duplication
Pp3c26_13600V3.1	Nucleus	
Pp3c15_24080V3.1	Nucleus	block duplication
Pp3c9_24170V3.1	Nucleus	block duplication
Pp3c13_5070V3.1	Nucleus	
Pp3c12_17180V3.1	Nucleus	
Pp3c4_12510V3.1	Nucleus	block duplication
Pp3c3_19360V3.1	Nucleus	block duplication
Pp3c1_42920V3.1	Nucleus	
Pp3c10_12410V3.1	Nucleus	
Pp3c8_16720V3.1	Nucleus	
Pp3c6_24650V3.1	Nucleus	
Pp3c5_2150V3.1	Nucleus	

Pp3c6_23360V3.1	Nucleus	
Pp3c5_7650V3.1	Nucleus	
Pp3c23_7530V3.1	Nucleus	block duplication
Pp3c20_8950V3.1	Nucleus	block duplication
Pp3c3_12080V3.1	Nucleus	
Pp3c1_1650V3.1	Nucleus	block duplication
Pp3c2_34670V3.1	Nucleus	block duplication
Pp3c10_10240V3.1	Nucleus	
Pp3c14_14030V3.1	Nucleus	
Pp3c12_10360V3.1	Nucleus	
Pp3c17_12730V3.1	Nucleus	
Pp3c15_19246V3.1	Nucleus	
Pp3c7_21220V3.1	Nucleus	block duplication
Pp3c4_13790V3.1	Nucleus	
Pp3c11_2650V3.1	Nucleus	block duplication
Pp3c18_340V3.1	Nucleus	
Pp3c3_17580V3.1	Nucleus	
Pp3c13_8880V3.1	Nucleus	
Pp3c24_11770V3.1	Nucleus	
Pp3c17_19947V3.1	Nucleus	
Pp3c23_18290V3.1	Nucleus	
Pp3c6_9970V3.1	Nucleus	tandem duplication
Pp3c13_1310V3.1	Nucleus	
Pp3c9_19090V3.1	Nucleus	
Pp3c15_23110V3.1	Nucleus	
Pp3c3_1630V3.1	Nucleus	
Pp3c15_23090V3.1	Nucleus	
Pp3c3_11280V3.1	Nucleus	
Pp3c2_20860V3.1	Nucleus	
Pp3c12_10900V3.1	Nucleus	
Pp3c4_5410V3.1	Nucleus	
Pp3c24_17130V3.1	Nucleus	
Pp3c9_19080V3.1	Nucleus	
Pp3c26_6730V3.1	Nucleus	
Pp3c1_19660V3.1	Nucleus	
Pp3c13_19170V3.1	Nucleus	
Pp3c4_16540V3.1	Nucleus	
Pp3c4_17230V3.1	Nucleus	
Pp3c20_1400V3.1	Nucleus	
Pp3c8_16730V3.1	Nucleus	
Pp3c7_11120V3.1	Nucleus	
Pp3c21_19330V3.1	Nucleus	
Pp3c11_23240V3.1	Nucleus	

Pp3c11_3110V3.1	Nucleus
Pp3c24_8050V3.1	Chloroplast. Nucleus.
Pp3s241_30V3.1	Nucleus
Pp3c11_8790V3.1	Nucleus
Pp3c18_14540V3.1	Nucleus
Pp3c14_14040V3.1	Nucleus
Pp3c12_8190V3.1	Nucleus
Pp3c20_1403V3.1	Nucleus
Pp3c20_23720V3.1	Chloroplast. Nucleus.
Pp3c5_29220V3.1	Nucleus
Pp3c19_23130V3.1	Nucleus
Pp3c12_21500V3.1	Nucleus
Pp3c21_11880V3.1	Nucleus
Pp3c11_8780V3.1	Nucleus
Pp3c7_16800V3.1	Nucleus
Pp3c2_32300V3.1	Nucleus
Pp3c7_20880V3.1	Nucleus
Pp3c4_2590V3.1	Nucleus
Pp3c18_8710V3.1	Nucleus
Pp3c7_2390V3.1	Nucleus
Pp3c12_1320V3.1	Golgi apparatus. Nucleus
Pp3c4_6990V3.1	Nucleus
Pp3c13_1060V3.1	Nucleus
Pp3c8_22450V3.1	Nucleus
Pp3s76_20V3.1	Nucleus
Pp3c4_7100V3.1	Nucleus
Pp3c15_19250V3.1	Nucleus
Pp3c2_40V3.1	Nucleus
Pp3c12_17450V3.1	Nucleus
Pp3c12_14300V3.1	Nucleus
Pp3c3_37540V3.1	Nucleus
Pp3c3_9330V3.1	Nucleus
Pp3c12_21290V3.1	Nucleus
Pp3c3_33220V3.1	Nucleus
Pp3c17_1730V3.1	Golgi apparatus. Nucleus
Pp3c14_5130V3.1	Golgi apparatus. Nucleus
Pp3c4_2120V3.1	Nucleus
Pp3c11_16920V3.1	Nucleus

Table S2. List of primers used in this study.

Gene name	Forward primer (5' to 3')	Reverse primer (5' to 3')
Pm999-6730	TAGCATCGATGCAATTCGGTAC CATGGCGGAATCCCAGTCCTCT	TCGCCCTTGCTCACCATTCTAGAATGAT GCATACCTGGCTGTGA
Pm999-8050	CTGACTCTAGCATCGATGAATT CGATGGATTTCGACATAA	GGCATGCACTAGTGTCGACTTCTAACGC TTGATCTACCCT
PM999-2590	GCATCGATGAATTCGGTACCC ATGAATGCCGCTAGTTCGG	CCCTTGCTCACCATTCTAGAAGTCCACC AAGTATCTACAC
Pm999-5410	CTGACTCTAGCATCGATGAATT CTATGGCTCGCGGGTGCT	CCCTTGCTCACCATTCTAGACACCACAC CGATCGCATTGC
